# Anti-tumor effects of anti-programmed cell death-1 antibody treatment are attenuated in streptozotocin-induced diabetic mice

**DOI:** 10.1038/s41598-023-33049-7

**Published:** 2023-04-12

**Authors:** Masaaki Ito, Shintaro Iwama, Daisuke Sugiyama, Yoshinori Yasuda, Takayuki Okuji, Tomoko Kobayashi, Xin Zhou, Ayana Yamagami, Takeshi Onoue, Takashi Miyata, Mariko Sugiyama, Daisuke Hagiwara, Hidetaka Suga, Ryoichi Banno, Hiroyoshi Nishikawa, Hiroshi Arima

**Affiliations:** 1 Department of Endocrinology and Diabetes, Nagoya University Graduate School of Medicine, Nagoya, 466-8550 Japan; 2 Department of Immunology, Nagoya University Graduate School of Medicine, Nagoya, 466-8550 Japan; 3 Research Center of Health, Physical Fitness and Sports, Nagoya University, Nagoya, 464-8601 Japan; 4 Division of Cancer Immunology, Research Institute/Exploratory Oncology Research & Clinical Trial Center (EPOC), National Cancer Center, Tokyo, 104-0045 Japan

**Keywords:** Diabetes, Chemokines, Tumour immunology, CD8-positive T cells, Dendritic cells

## Abstract

Hyperglycemia impairs immune response; however, it remains unknown whether the anti-tumor effects of anti-programmed cell death-1 antibody (PD-1-Ab) treatment are changed in hyperglycemic conditions. We analyzed the effect of PD-1-Ab on tumor growth in streptozotocin-induced diabetic mice (STZ-mice) subcutaneously inoculated with MC38 (a colon carcinoma cell line). Furthermore, we assessed the expression of chemokines by polymerase chain reaction (PCR) array in tumor-draining lymph nodes (dLNs) of these mice and MC38 cells cultured in different glucose concentrations. The suppressive effect of PD-1-Ab on tumor growth was attenuated. This was accompanied by fewer tumor-infiltrating CD8^+^ T cells, and STZ-mice had fewer tumor-infiltrating CD11c^+^ dendritic cells (DCs) than normoglycemic mice. mRNA expression levels of CXCL9, a chemokine recruiting CD8^+^ T cells, were lower in dLNs of STZ-mice than in normoglycemic mice after PD-1-Ab treatment, and its protein was expressed in DCs. In MC38 cells cultured with 25 mM glucose, mRNA expression of CCL7, a chemokine recruiting DCs, was decreased compared to cells cultured with 5 mM glucose. These results suggest that the STZ-induced hyperglycemia impairs the effect of PD-1-Ab treatment on MC38 tumor growth, and is accompanied by reduced infiltration of DCs and CD8^+^ T cells and decreased expression of CCL7 and CXCL9.

## Introduction

Relative risks of developing various malignancies, including colorectal cancer, are increased in patients with diabetes mellitus^1^. The risk of colorectal cancer is associated with increased levels of random^2^ and fasting blood glucose^3^. Furthermore, a meta-analysis reported that diabetes mellitus is associated with cancer-specific mortality in patients with colorectal cancer^4^, suggesting that diabetes may be a poor prognostic factor in patients with colorectal cancer.

Diabetes is also well known to cause immune dysfunction. In addition to dysfunction of the innate immune system^5^, the diabetic condition impairs acquired immune responses by T cells^6,7^. Infiltration of CD8^+^ T cells into malignant melanoma lesions was found to be decreased in streptozotocin (STZ)-induced diabetic mice^8^ and the proliferative capacity of splenic T cells was decreased in STZ-induced diabetic rats^9^. Since T cells play an important role in anti-tumor immunity^10^, impaired anti-tumor T cell responses may partially explain the increased risk of malignancy in diabetes.

Immune checkpoint inhibitors (ICIs), including antibodies against programmed cell death-1 (PD-1) and PD-1 ligand-1 (PD-L1), induce their anti-tumor effects by improving the function of immune cells^11^. Two retrospective studies from Israel^12^ and Japan^13^ recently reported that diabetes was negatively correlated to overall survival in patients with non-small cell lung cancer (NSCLC) treated with ICIs. However, the mechanisms behind this impaired outcome of ICI treatments in diabetic patients remain unknown.

To investigate the effect of hyperglycemia on anti-tumor function induced by anti-PD-1 antibody treatment, we treated STZ-induced diabetic mice with subcutaneous MC38 colon carcinoma tumors with anti-PD-1 antibody, and examined changes in tumor growth, the number of tumor-infiltrating immune cells, and the expression of chemokines and chemokine receptors.

## Results

### The effects of anti-PD-1 antibody on tumor growth were attenuated in STZ-induced diabetic mice

**Figure 1:**
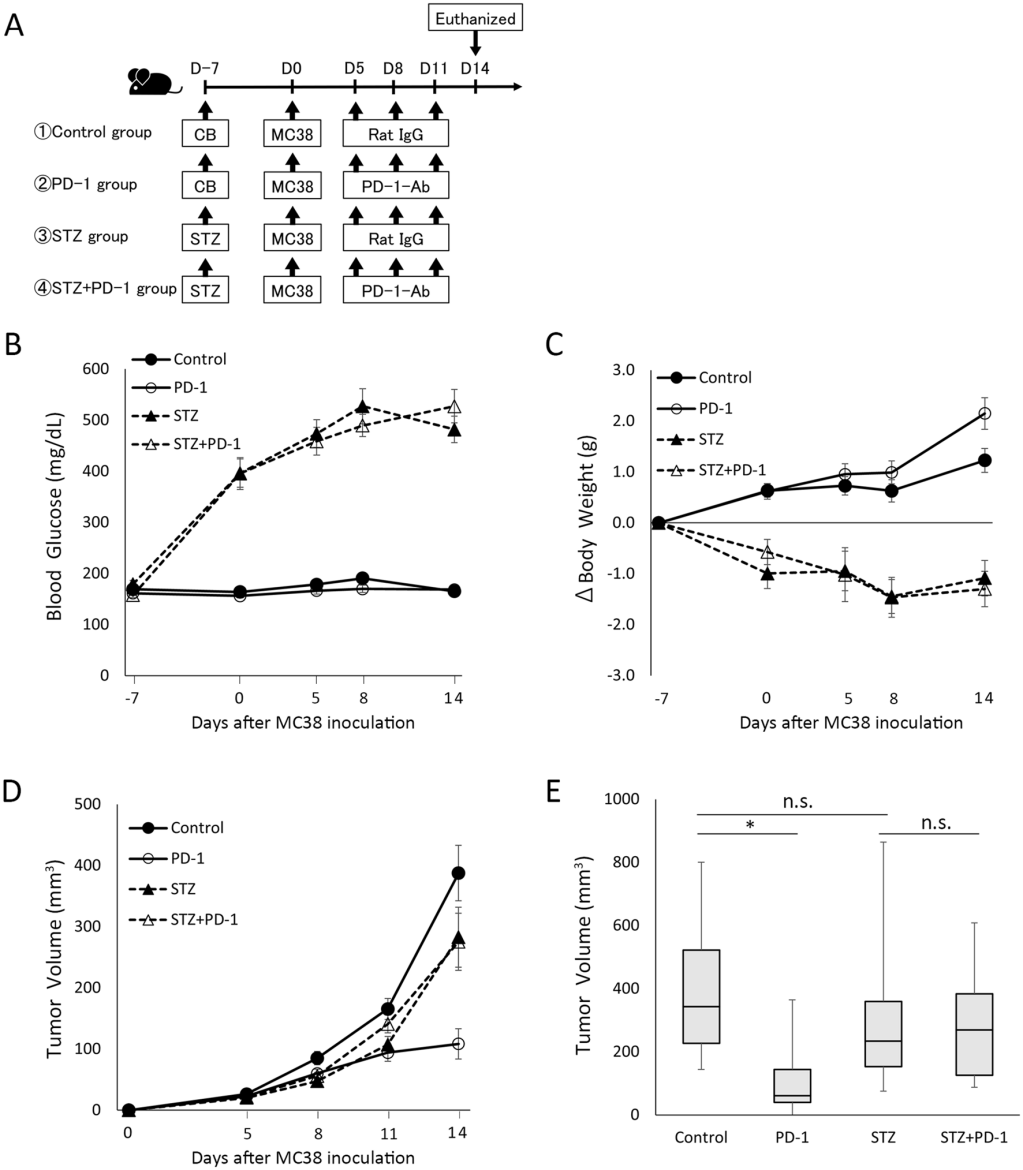
The effect of anti-PD-1 antibody on MC38 tumor is attenuated in STZ-induced diabetic mice. (**A**) Experimental timeline examining the effect of anti-PD-1 antibody on MC38 tumor growth in STZ-induced diabetic mice. Control group (n = 18), PD-1 group (n = 20), STZ group (n = 15), and STZ + PD-1 group (n = 11). (**B**) Blood glucose levels in the control, PD-1, STZ, and STZ + PD-1 groups. (**C**) Changes in body weight in the control, PD-1, STZ, and STZ + PD-1 groups. (**D**) Tumor volume in the control, PD-1, STZ, and STZ + PD-1 groups. (E) Box and whisker plots of tumor volume on day 14 in control, PD-1, STZ, and STZ + PD-1 groups. Boxes indicate median values with interquartile range, and whiskers indicate the lowest and highest values. Graphical data from (**B–D**) are presented as mean ± SEM. Kruskal–Wallis and Mann–Whitney U tests with Bonferroni correction are used in (**E**). *P < 0.05. *Ab *antibody, *CB* sodium citrate buffer, *n.s.* not significant, *PD-1* programmed cell death-1, *SEM* standard error of the mean, *STZ* streptozotocin.

To induce hyperglycemia, mice were intraperitoneally injected with STZ, which can damage pancreatic β cells resulting in the development of insulin dependent diabetes, a model of type 1 diabetes mellitus (T1DM). Mice were intraperitoneally injected with STZ or sodium citrate buffer (CB) 7 days before subcutaneous inoculation with MC38 cells in the right flank on day 0 (Fig. 1A). Then, the mice were treated with anti-PD-1 antibody or normal rat IgG on days 5, 8, and 11. The anti-tumor effects of anti-PD-1 antibody treatment were examined in the following four groups: mice injected with CB followed by normal rat IgG (control group), mice injected with CB followed by anti-PD-1 antibody (PD-1 group), mice injected with STZ followed by normal rat IgG (STZ group), and mice injected with STZ followed by anti-PD-1 antibody (STZ + PD-1 group) (Fig. 1A). Serum glucose levels were significantly increased in the STZ and STZ + PD-1 groups compared with the control and PD-1 groups (Fig. 1B). The body weights were significantly lower in the STZ and STZ + PD-1 groups than in the control and PD-1 groups (Fig. 1C). After subcutaneous inoculation with MC38 cells, tumor volume in each mouse was measured on days 5, 8, 11, and 14 (Fig. 1D). Treatment with anti-PD-1 antibody significantly reduced the tumor volumes in the PD-1 group as compared to the control group on day 14 (Fig. 1D,E). In contrast, the reduction of tumor volume was not observed in the STZ + PD-1 group, compared to the STZ group (Fig. 1D,E).

### The number of tumor-infiltrating CD8^+^ T cells did not increase after anti-PD-1 antibody treatment in STZ-induced diabetic mice

**Figure 2:**
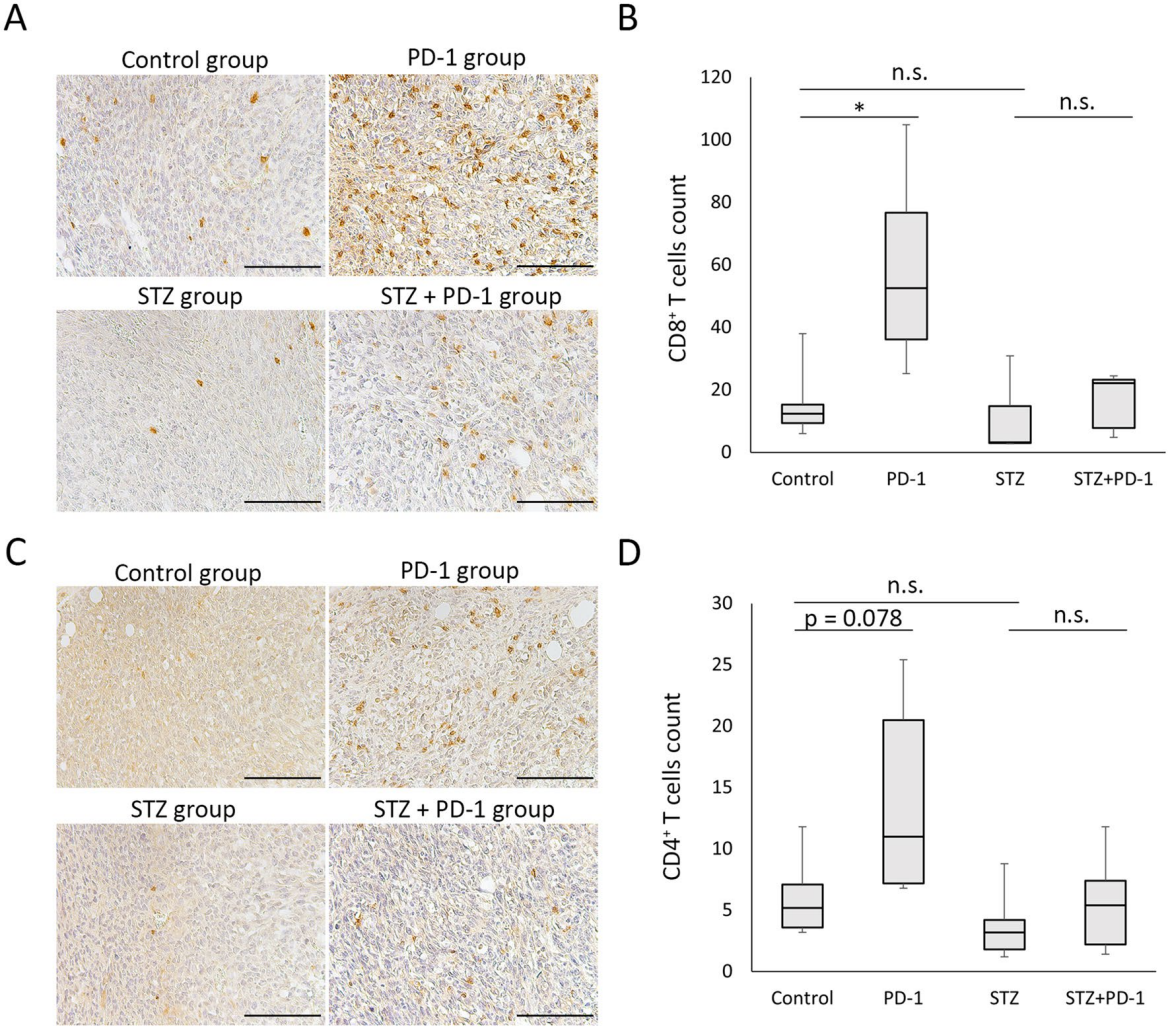
The number of tumor-infiltrating CD8^+^ and CD4^+^ T cells after anti-PD-1 antibody treatment is decreased in STZ-induced diabetic mice. Immunohistochemical staining of CD8^+^ (**A**) and CD4^+^ T cells (**C**) in MC38 tumor in the control, PD-1, STZ, and STZ + PD-1 groups. Scale bars, 100 µm. The number of CD8^+^ (**B**) and CD4^+^ T cells (**D**) in MC38 tumors in control (n = 6), PD-1 (n = 6), STZ (n = 5), and STZ + PD-1 (n = 5) groups. Boxes indicate median values with interquartile range, and whiskers indicate the lowest and highest values. Kruskal–Wallis and Mann–Whitney U tests with Bonferroni correction are used in (**B**), and one-way ANOVA and Tukey’s test are used in (**D**). *P < 0.05. *ANOVA* analysis of variance, *n.s.* not significant, *PD-1* programmed cell death-1, *STZ* streptozotocin.

Next, we examined the number of tumor-infiltrating T cells by immunohistochemical staining of CD4 or CD8. The number of tumor-infiltrating CD8^+^ T cells was significantly increased in the PD-1 group compared with the control group on day 14 (Fig. 2A,B). In contrast, this increase in tumor-infiltrating CD8^+^ T cells was not observed in the STZ + PD-1 group when compared to the STZ group (Fig. 2A,B). Similarly, the number of tumor-infiltrating CD4^+^ T cells tended to be increased (p = 0.078) in the PD-1 group as compared to the control group on day 14 (Fig. 2C,D). However, there was no difference in the number of tumor-infiltrating CD4^+^ T cells between the STZ and STZ + PD-1 groups (Fig. 2C,D).

### STZ-induced diabetes did not change the frequency of memory T cell subsets, PD-1H^+^ T cells, or the mRNA expression of PD-L1 in tumors

The frequencies of memory T cell subsets and that of PD-1H^+^ cells in the total CD4^+^ and CD8^+^ T cell pools isolated from tumor-draining lymph nodes (dLNs) were analyzed by flow cytometry. There was no difference in the frequency of memory T cell subsets [central memory (CD44^+^ CD62L^+^) and effector memory (CD44^+^ CD62L^−^) T cells] or PD-1H^+^ cells in the CD8^+^ and CD4^+^ T cell pools among the four groups (Supplementary Figs. S1 and S2). Using quantitative real-time reverse transcriptase polymerase chain reaction (qRT-PCR), we did not detect any changes in the mRNA expression of PD-L1 in MC38 tumors among the four groups (Supplementary Fig. S3).

### mRNA expression levels of CCL7 decreased in MC38 cells cultured with high glucose concentrations compared to low glucose concentrations

**Figure 3:**
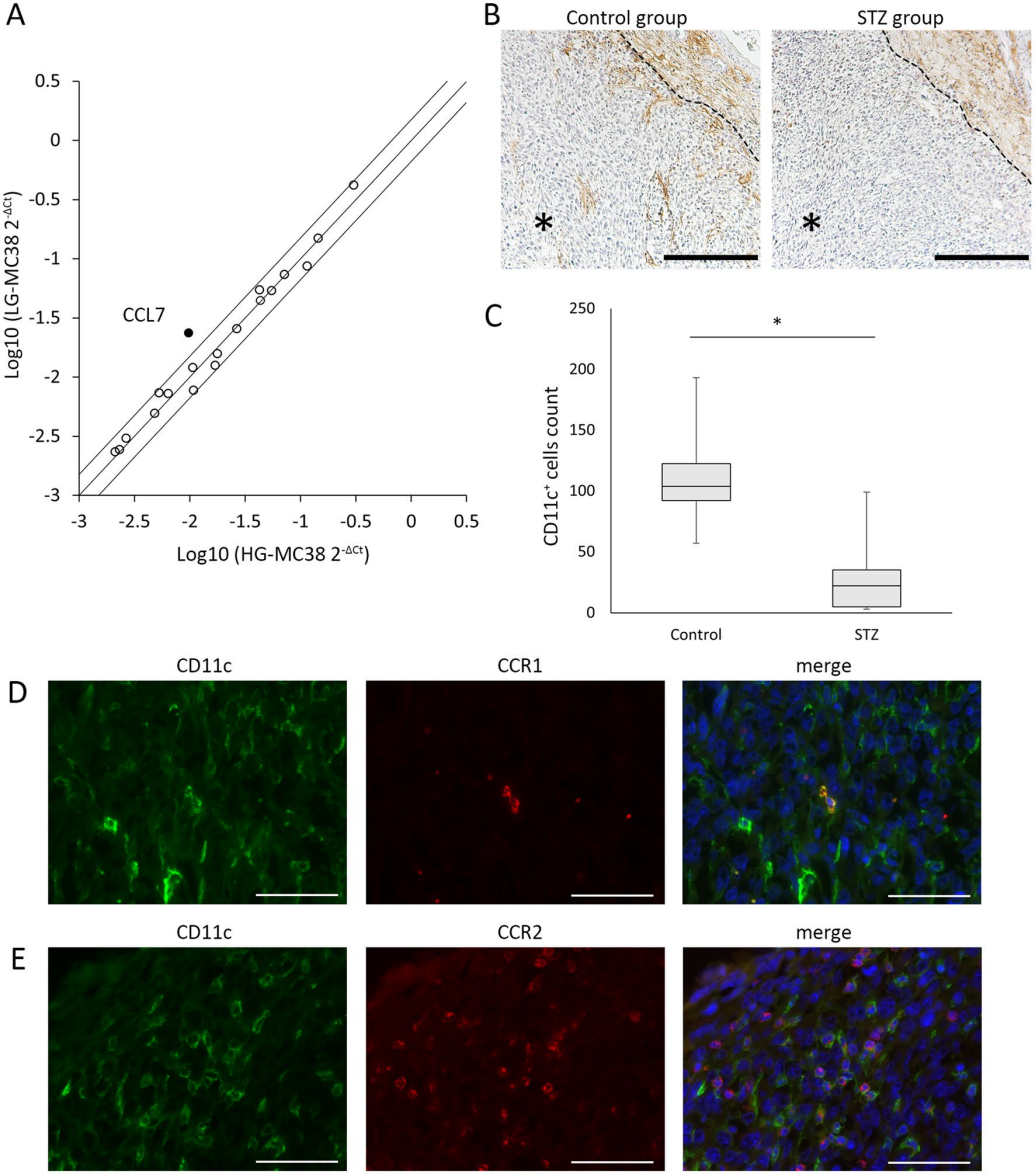
The effect of hyperglycemia on the expression of chemokines and chemokine receptors in MC38 cells and the number of tumor-infiltrating CD11c^+^ cells. (**A**) Scatterplot of mRNA expression levels of chemokines and chemokine receptors in MC38 cells cultured with low glucose media (LG-MC38) or with high glucose media (HG-MC38) analyzed by the RT^2^ Profiler PCR Array. The middle line indicates that the expression levels are equal between LG-MC38 and HG-MC38. The upper and lower lines indicate a 1.5-fold increase or decrease in LG-MC38 compared to HG-MC38. (**B**) Immunohistochemical staining of CD11c^+^ cells in MC38 tumors in the control or STZ groups. Scale bars, 200 µm. The dashed line indicates the edge of the tumor. Asterisk: tumor tissue area. (**C**) The number of CD11c^+^ cells in MC38 tumors in the control and STZ groups. Boxes indicate median values with interquartile range, and whiskers indicate the lowest and highest values. (**D,E**) Double immunofluorescence microscopy shows the expression of CCR1 (red) (**D**) or CCR2 (red) (**E**) on CD11c^+^ cells (green) in MC38 tumor, respectively. Scale bars, 50 µm. Kruskal–Wallis and Mann–Whitney U tests with Bonferroni correction are used in (**C**). *P < 0.05. *STZ* streptozotocin.

To investigate the effect of hyperglycemia on chemokine expression in MC38 cells, we examined the expression levels of 84 genes for chemokines and chemokine receptors in MC38 cells cultured with low glucose (5 mM) or high glucose (25 mM) concentrations (LG-MC38 and HG-MC38) by an RT^2^ Profiler PCR Array. The PCR array analysis found 19 genes with Ct values under 30 in both the LG-MC38 and HG-MC38 groups (Supplementary Table S1). Among these 19 genes, CCL7 was the only gene showing more than 1.5 times higher mRNA expression levels in the LG-MC38 group than in the HG-MC38 group (fold change; 2.40) (Fig. 3A). In MC38 tumors extracted from mice, there was no difference in CCL7 mRNA expression among the four groups (Supplementary Fig. S4).

### The number of tumor-infiltrating CD11c^+^ cells was decreased in STZ-induced diabetic mice

Since CCL7 is known to be involved in the migration of dendritic cells (DC) into tumors^14^, the number of tumor-infiltrating DCs was analyzed in MC38 tumor tissues at day 14 by using CD11c as a marker for DCs. Although CD11c^+^ cells were present in both the control and the STZ groups around the tumors, as assessed by immunohistochemistry (Fig. 3B), the number of tumor-infiltrating CD11c^+^ cells was significantly lower in the STZ than in the control groups (Fig. 3B,C). In contrast, there were no differences in the number of tumor-infiltrating CD11c^+^ cells between control and PD-1 groups, or between STZ and STZ + PD-1 groups (Supplementary Figs. S5 and S6). Double immunofluorescence microscopy showed that tumor-infiltrating CD11c^+^ cells were positive for CCR1 and CCR2 (receptors of CCL7) in the control group (Fig. 3D,E). On the other hand, there were no differences in the number of CD11c^+^ cells in dLNs among the four groups (Supplementary Fig. S7).

### mRNA expression levels of CXCL9 in dLNs was decreased in STZ-induced diabetic mice treated with anti-PD-1 antibody

**Figure 4:**
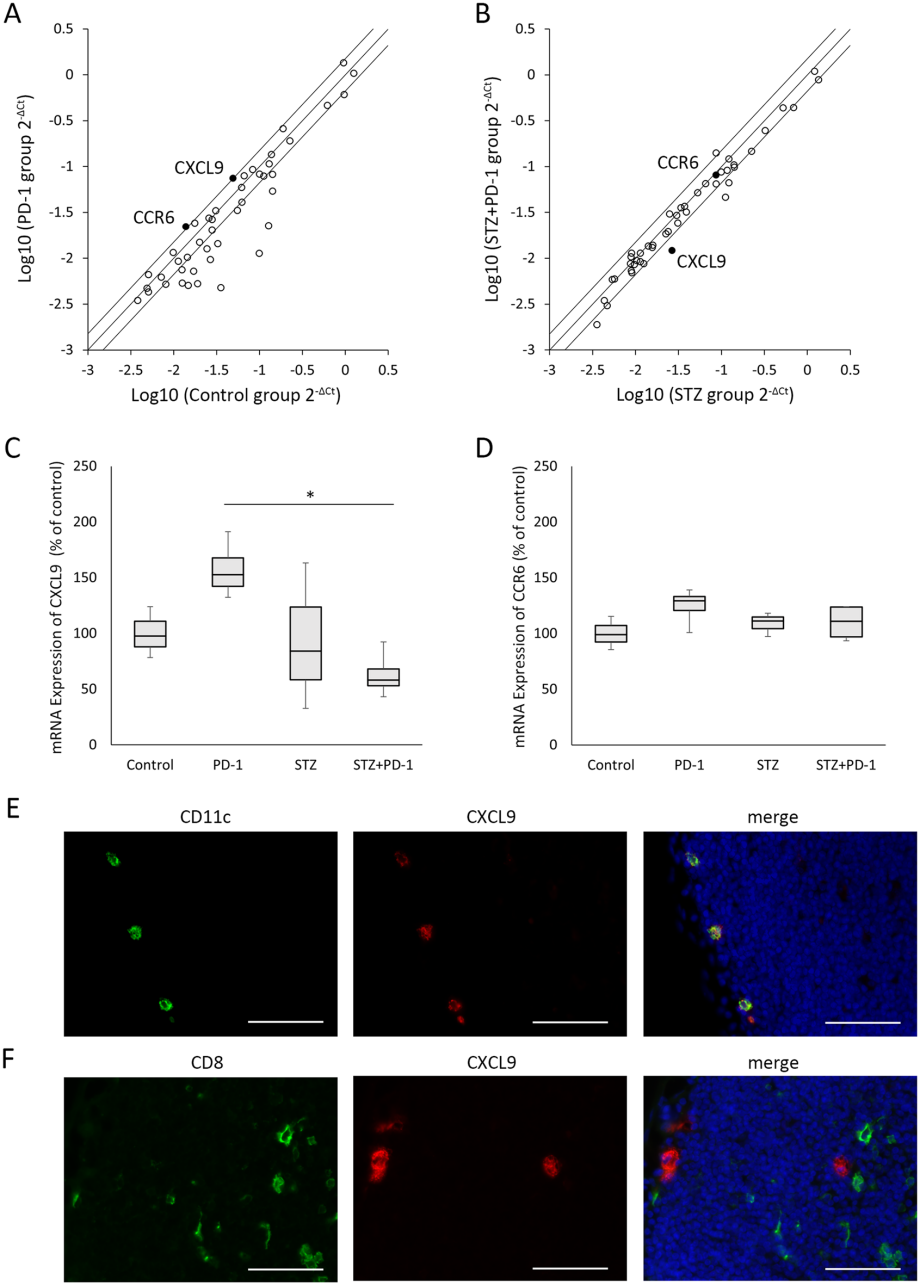
The mRNA expression levels of chemokines and chemokine receptors in dLNs from STZ-induced diabetic mice. (**A,B**) Scatterplot of mRNA expression levels of chemokines and chemokine receptors in dLNs of the control and PD-1 groups (**A**) and the STZ and STZ + PD-1 groups (**B**) analyzed by RT^2^ Profiler PCR Array. The middle line indicates that the expression levels are equal between the two groups. The upper and lower lines indicate a 1.5-fold increase or decrease in the two groups. (**C,D**) The mRNA expression levels of CXCL9 (**C**) and CCR6 (**D**) as assessed by qRT-PCR in dLNs. Boxes indicate median values with the interquartile range, and whiskers indicate the lowest and highest values. One-way ANOVA and Tukey’s test were used in (**C,D**). *P < 0.05. (**E,F**) Double immunofluorescence microscopy shows the expression of CXCL9 (red) on CD11c^+^ cells (green) (**E**) or CD8^+^ cells (green) (**F**) in dLNs, respectively. Scale bars, 50 µm. *ANOVA* Analysis of variance, *CCR6* C–C motif chemokine receptor 6, *CXCL9* C-X-C motif chemokine ligand 9, *dLNs* tumor-draining lymph nodes, *PD-1* programmed cell death-1, *qRT-PCR* quantitative real-time reverse transcriptase polymerase chain reaction, *STZ* streptozotocin.

To identify genes that contributed to the recruitment of CD8^+^ T cells into dLNs and were affected by the STZ-induced diabetic condition, the RT^2^ Profiler PCR Array analysis was performed in dLNs from mice in the four groups. Among the 84 genes investigated, 45 genes were found to have a Ct value under 30 in the four groups (Supplementary Table S2 and S3). In this analysis, we focused on the genes that were upregulated more than 1.5 times in the PD-1 group, compared to the control groups (Fig. 4A), but were not upregulated in the STZ + PD-1 group, as compared to the STZ groups (Fig. 4B). We found that CXCL9 (fold change; 1.59) and CCR6 (fold change; 1.52) met these criteria. Using qRT-PCR, we found that the mRNA expression of CXCL9 was significantly decreased in the STZ + PD-1 group, as compared to the PD-1 group (p = 0.018), although there was no statistical difference in the CXCL9 expression between the PD-1 and control groups (p = 0.222) (Fig. 4C). On the other hand, we did not detect a difference in the expression of CCR6 among the four groups using qRT-PCR analysis (Fig. 4D). Immunohistochemical analyses revealed that CXCL9 was expressed in CD11c^+^ cells in the subcapsular sinus and interfollicular areas (Fig. 4E) (Supplementary Fig. S8), but not in CD8^+^ T cells in interfollicular areas (Fig. 4F) (Supplementary Fig. S8), in dLNs from mice bearing MC38 tumors.

## Discussion

In this study, we showed that the suppression of tumor growth and the increased number of tumor-infiltrating CD8^+^ T cells after anti-PD-1 antibody administration in normoglycemic mice was not detected in STZ-induced diabetic mice. Since it was previously reported that PD-1 blockade increases the number of tumor-infiltrating CD8^+^ T cells in mice bearing MC38 tumors^15^, and the number of tumor-infiltrating CD8^+^ T cells was associated with improved prognosis^16–18^, tumor-infiltrating CD8^+^ T cells are thought to have an important role in the effects of anti-PD-1 antibody treatment. These data suggest that the STZ-induced diabetic condition interfered with the migration of CD8^+^ T cells into MC38 tumors, resulting in impaired effects of anti-PD-1 antibody treatment on tumor growth in our mouse model.

Chemokines are a group of cytokines whose main function is to induce the migration of leukocytes that can move based on the gradient of chemokine concentrations^19^. In this study, CXCL9 expression in dLNs was lower after the administration of anti-PD-1 antibody in STZ-induced diabetic mice than in normoglycemic mice. CXCL9, also known as monokine induced by gamma interferon (MIG), is known to have important roles in anti-tumor immunity, especially in the recruitment of T cells^20^. For example, it was reported that marked infiltration of T cells was exclusively detected in tumor lesions where CXCL9 was strongly expressed in patients with malignant melanoma^21^. CXCL9 is thought to be a key chemokine in anti-PD-1 antibody treatment, since the number of tumor-infiltrating CD8^+^ T cells induced by anti-PD-1 antibody treatment was lower in both CXCL9 knockout and CXCR3, the receptor of CXCL9, knockout mice bearing MC38 tumors, resulting in an impaired effect on tumor growth^22^. It was reported that myeloid cells, including tumor-associated macrophages and DCs, in peripheral blood mononuclear cells (PBMCs) secrete CXCL9 in patients with epithelial ovarian cancer^23^. Furthermore, high expression levels of CXCL9 and CCL5 were associated with an increased number of tumor-infiltrating CD8^+^ T cells and better outcomes after PD-1 inhibition in not only patients with ovarian cancer, but also those with other cancers, including colon, lung, and breast cancer^23^. The role of CXCL9 in dLNs is not fully understood in anti-tumor immunity; although CXCL9 has been reported to play an essential role in recruiting central memory T cells to the periphery of lymph nodes and induce recall response of CD8^+^ T cells in mice infected with lymphocytic choriomeningitis virus^24^. Since CXCL9 was expressed in CD11c^+^ cells, but not in CD8^+^ T cells, it was suggested that the STZ-induced diabetic condition interferes with the increase of CXCL9 expression detected after anti-PD-1 antibody treatment in CD11c^+^ cells, which may explain the impaired effect of anti-PD-1 antibody treatment on tumor growth and the decreased number of tumor-infiltrating CD8^+^ T cells in this model. In addition, the effect of STZ on systemic blood cells should be investigated further in future studies, as there is at least one report showing a decrease in the number of T cells following STZ injection in mice^25^.

In this study, we showed that the expression levels of CCL7 were decreased in MC38 cells cultured with high glucose concentrations, the number of tumor-infiltrating CD11c^+^ cells decreased in STZ-induced diabetic mice, and that CCR1 and 2 were expressed on tumor-infiltrating CD11c^+^ cells, which may be another mechanism involved in the reduced anti-tumor efficacy of anti-PD-1 antibody in this model. CCL7, also known as monocyte chemotaxis protein 3 (MCP-3), acts on various leukocytes, including DCs, which play a central role in antigen-specific immunity^26^. It was reported that T cell-dependent anti-tumor immunity was activated in mice transplanted with the mastocytoma cell line (P815) transfected with a gene encoding CCL7. In this model, DCs accumulated in peritumoral tissues, resulting in the suppression of tumor growth^27^. Another study using mice transplanted with colorectal cancer cells (CMT93) transfected with a gene encoding CCL7 also showed tumor growth reduction^28^. In a mouse lung cancer model, CCL7 deficiency resulted in impaired anti-tumor effects of CD8^+^ T cells and decreased infiltration of DCs into tumor tissues^14^. In contrast, the anti-tumor effects of anti-PD-1 antibody treatment were enhanced by the induction of the CCL7 gene into the lung tumor using a Lenti virus vector^14^. Moreover, in patients with NSCLC, the higher expression of CCL7 in tumor tissues was positively correlated with an increased number of tumor-infiltrating DCs and better overall survival^14^. Taken together, we suggest that the decreased expression of CCL7 by MC38 cells induced by hyperglycemia resulted in reduced tumor infiltration of DCs, resulting in limited CD8^+^ T cell tumor infiltration due to a lack of cancer antigen presentation by DCs in the dLNs. As the number of CD11c^+^ cells in dLNs was unchanged, the decreased expression of CXCL9 could have been caused by impaired function of CD11c^+^ cells rather than an decreased number of cells. Since there was no difference in CCL7 expression in vivo, possibly due to the MC38 tumor exhibiting a variety of cells, it is important to analyze the change in CCL7 expression in MC38 cells isolated from subcutaneous tumors.

Although we did not detect any differences in the frequency of effector and central memory T cell subsets among CD8^+^ and CD4^+^ T cells in the four groups in this study, changes in the frequencies of these T cell subsets have been detected in patients undergoing PD-1 blockade^29^ and diabetic patients^30^. For example, it was reported that the frequency of effector memory T cells specific for tumor antigen was increased in mice showing a better response to anti-PD-1 antibody treatment for malignant mesothelioma^29^ and decreased frequencies of naïve T cells and increased frequencies of exhausted T cell subsets were observed in patients with type 2 diabetes^30^. Although the effect of hyperglycemia on the anti-tumor effects induced by PD-1 antibody treatment should be examined in mice with other tumors in the future, our data showing no changes in the frequency of these T cell subsets suggests that impaired migration, rather than alterations in effector T cell subsets, causes the reduced anti-tumor efficacy of anti-PD-1 antibody treatment in STZ-induced diabetic mice.

We did not detect any differences in the expression of PD-1H (a marker for inactivation of CD8^+^ T cells^31^) on T cells or PD-L1 on MC38 cells between the STZ-induced diabetic and normoglycemic mice in this study. PD-1 expression is also known to be another marker for inactivation of CD8^+^ T cells. Although a meta-analysis of five studies reported that PD-1 expression was increased in CD4^+^ and CD8^+^ T cells in PBMCs from diabetic patients in comparison to controls, the result in each study varied among the five studies, with two studies showing increased levels, two studies showing comparable levels, and one study showing decreased levels of PD-1 expression in CD4^+^ T cells in patients with type 2 diabetes^32^. Another study reported that PD-1 expression on CD4^+^ and CD8^+^ T cells was not different between patients with type 2 diabetes and healthy controls^33^. In addition, it may be difficult to evaluate PD-1 expression in mice injected with anti-PD-1 antibody due to a possibility of internalization of PD-1 and competition of detection antibody with injected antibody. As for PD-L1 expression in tumor tissues, only one study reported higher PD-L1 expression as assessed by immunohistochemistry in patients with NSCLC and diabetes than in patients who did not have diabetes^34^. Taken together, it remains unclear if hyperglycemia is associated with changes in expression patterns of PD-1 and PD-L1, because most studies analyzed patients with type 2 diabetes, in whom the data may be affected by hyperglycemia and their chronic inflammatory condition due to obesity.

In this study, we analyzed the effect of anti-PD-1 antibody on tumor growth in STZ-induced diabetic mice, a model of acute onset T1DM. It is well known that T1DM can occur with anti-PD-1 antibody treatment, although the incidence is relatively low (less than 1%)^35^. Therefore, the impaired effect of anti-PD-1 antibody on tumor growth found in this study may be generalized to patients who develop acute T1DM during anti-PD-1 antibody treatment. Although it was reported that development of endocrine irAEs, such as pituitary dysfunction and thyroid dysfunction^36^, was associated with better overall survival, it should be interesting to analyze overall survival in patients who develop T1DM induced by ICIs in the future. It will also be important to evaluate whether the anti-tumor effect of anti-PD-1 antibody can be changed under hyperglycemic conditions due to type 2 diabetes.

This study has some limitations. First, findings using STZ-induced diabetic mice may not completely reflect the pathogenesis in diabetic patients. Second, the mechanisms by which the decreased number of CD11c^+^ cells in tumors leads to a reduction in tumor-infiltrating CD8^+^ T cells in STZ-induced diabetic mice remain unknown. Third, it also remains unclear if impaired effects of anti-PD-1 antibody treatment on tumor growth may be reversed with treatment for hyperglycemia. If this is the case, the treatment of diabetes will have high importance for cancer immunotherapy using anti-PD-1 antibody therapy in the future. Fourth, flow cytometric analyses were not performed in subcutaneous lymph nodes from mice without MC38 tumors, so any differences in the number of cells in the lymph nodes between normal and STZ-mice were not evaluated.

In conclusion, the anti-tumor effects induced by anti-PD-1 antibody treatment were found to be attenuated in STZ-induced diabetic mice, which was accompanied by decreased infiltration of CD11c^+^ cells and CD8^+^ T cells and decreased expression of CCL7 and CXCL9.

## Methods

### Cell culture

MC38, a mouse colon carcinoma cell line of C57BL/6 origin, was obtained from Kerafast (#ENH204, RRID: B288). MC38 cells were maintained in Dulbecco's Modified Eagle’s medium (DMEM) with high glucose concentration (25 mM) supplemented with 10% fetal bovine serum (FBS), 0.1 mM non-essential amino acids, 1 mM sodium pyruvate, 10 mM 4-(2-hydroxyethyl)piperazine-1-ethanesulfonic acid (HEPES), 50 units/ml penicillin, and 50 μg/ml streptomycin. MC38 cells were cultured with low glucose (5 mM) or high glucose (25 mM) concentrations for 14 days (LG-MC38 and HG-MC38).

### Mice

Male C57BL/6J mice were purchased from Charles River Laboratories (Yokohama, Japan) and used for experiments at 7 weeks of age. All mice were maintained as described previously^37^. STZ-induced diabetic mice were created by a single intraperitoneal administration of 150 mg/kg STZ (Sigma-Aldrich) diluted with 0.09 M CB on day -7. Control mice received a single intraperitoneal administration of 0.09 M CB as the vehicle on day -7. Glucose levels were measured in blood from tail veins by a validated one-touch basic glucose measurement system (Glutest mint, Sanwa Kagaku Kenkyusho CO., LTD.). Body weight was measured on days -7, 0, 5, 8, and 14. If blood glucose levels were < 300 mg/dl 2 days after the first STZ administration (day -5), the mice were readministrated with the same dose of STZ intraperitoneally. Mice showing blood glucose levels < 300 mg/dl on day 0 or weight loss > 3 g from baseline were excluded from the analyses. All animal procedures were approved by the Animal Care and Use Committee of Nagoya University Graduate School of Medicine and performed in accordance with the institutional guidelines that conform to the National Institutes of Health animal care guidelines. The study was carried out in compliance with the ARRIVE guidelines.

### Tumor growth assay

Seven days after intraperitoneal administration of STZ or CB (day 0), 1 × 10^6^ MC38 cells were subcutaneously injected into the right flank of the mice. On days 5, 8, and 11, mice were injected intraperitoneally with rat anti-mouse PD-1 antibody (200 μg/each) (#BE0146, clone: RMP1-14, Bio X Cell) or normal rat IgG (#147-09521, WAKO) as a control. Tumor diameters (mm^2^) were measured by a caliper on days 5, 8, 11, and 14, and then tumor volumes (mm^3^) were calculated using the following formula: volume = [length (mm) × width^2^ (mm)]/6. On day 14, tumor tissues and dLNs were obtained from all mice. A dLN in the subcutaneous tissue of the right flank was confirmed by detecting dye flow 24 h after injection of 0.4% trypan blue into the MC38 tumor (Supplementary Fig. S9). The dose of anti-PD-1 antibody was determined based on previous studies^38,39^.

### Immunohistochemistry

MC38 tumors and dLNs obtained from mice on day 14 were treated with Beckstead’s zinc fixative^40^ and then processed as described previously^41^. Fixed tissues were cut into 4 µm sections, and stained by immunohistochemistry (#CTS017, R&D Systems) or immunofluorescence staining, as described previously^42,43^. The following primary antibodies were used in this study; anti-CD4 (#HS-360 004, Synaptic Systems), anti-CD8a (#14-0808-82, Invitrogen), anti-CD11c (#ab52632, Abcam, #14-0114-82, Invitrogen), anti-CCR1 (#NBP1-78173, NOVYS Biologicals), anti-CCR2 (#ab273050, abcam), and anti-CXCL9 (#AF-492, R&D Systems). Immunofluorescence was observed with a fluorescence microscope (BZ-9000 or BZ-X800, Keyence).

### Counts for tumor-infiltrating cells

Images taken with an optical microscope were used to count the number of tumor-infiltrating CD4^+^, CD8^+^, and CD11c^+^ cells. Images of five independent fields of MC38 tumor tissue (200× magnification) were used to count tumor-infiltrating CD4^+^ and CD8^+^ cells. Images of the marginal area of the MC38 tumor with the most abundant positive cells (200× magnification) were used to count tumor-infiltrating CD11c^+^ cells.

### Flow cytometry

Single-cell suspensions were obtained from the dLNs of mice, as described previously. Cells were rinsed with PBS and incubated with Fc block (#101320 and #422302, BioLegend) for 10 min at 4 °C to decrease the background signal. After washing, cells were stained with anti-CD3 (#100203, BioLegend), anti-CD4 (#116019, BioLegend), anti-CD8 (#100750, BioLegend), anti-CD44 (#103007, BioLegend), anti-CD62L (#104418, BioLegend), and anti-PD-1H (#143715, BioLegend) for 15 min at 4 °C, and a Live/Dead stain (#65-0866, Invitrogen) to exclude dead cells. All data from flow cytometric analyses (LSR Fortessa X-20, BD Biosciences) were analyzed using FlowJo software (BD Biosciences). In flow cytometry experiments, total counts up to 300,000 were analyzed in most samples.

### qRT-PCR

Total RNA was extracted from dLNs and tumor tissues from mice and cultured MC38 cells (RNeasy kit, Qiagen)^41^. cDNA was synthesized from 500 ng of total RNA using ReverTra Ace qPCR RT Kit (Toyobo). The qRT-PCR reactions were performed using Brilliant III Ultra-Fast SYBR Green qPCR Master Mix (Agilent Technologies), and samples were run using CFX Connect (Bio-Rad). GAPDH was used as an internal control to assess the relative mRNA levels, which were calculated using the comparative Ct method, as described previously^41^. The sequences of primers used were: CXCL9, forward: 5′-CCGAGGCACGATCCACTAC, reverse: 5′-CCGGATCTAGGCAGGTTTG; CCR6, forward: 5′-TTGTCCTCACCCTACCGTTC, reverse: 5′-GATGAACCACACTGCCACAC; PD-L1, forward: 5′-GCGGACTACAAGCGAATCAC, reverse: 5′-CTCAGCTTCTGGATAACCCTCG; GAPDH, forward: 5′- TGCACCACCAACTGCTTAG, reverse: 5′- GGATGCAGGGATGATGTTC.

### RT^2^ profiler PCR array

The RT^2^ Profiler™ PCR Array (#PAMM-022Z, QIAGEN, https://geneglobe.qiagen.com/us/product-groups/rt2-profiler-pcr-arrays) was used to examine the expression levels of a panel of 84 chemokine or chemokine receptor genes. Total RNA was extracted from MC38 cells (LG-MC38 and HG-MC38), and dLNs and tumor tissues from mice (n = 3–4/group), and then reverse transcribed to cDNA. The expression levels of 84 genes were analyzed according to the manufacturer’s instructions. Fold changes in gene expression were analyzed by using the web-based analysis tool of the GeneGlobe Data Analysis Center (https://dataanalysis2.qiagen.com/pcr). Genes with CT values over 30 were excluded.

### Statistical analysis

In all experiments, we first tested for normality with the Shapiro–Wilk test. When the population followed a normal distribution, the two-tailed Student's t-test was used to compare two groups, and one-way ANOVA and Tukey’s test were used to compare multiple groups. When the population did not follow a normal distribution, the Mann–Whitney U test was used to compare two groups, and the Kruskal–Wallis and Mann–Whitney U tests with Bonferroni correction were used to compare multiple groups. Graphical data are presented as means ± SEM. In box and whisker plots, boxes indicate median values with the interquartile range and whiskers indicate the lowest and highest values. P < 0.05 was considered statistically significant in all cases. Statistical analyses were performed with IBM SPSS Statistics V.28.

## Supplementary Information

Supplementary Information.

## Data Availability

The datasets generated and/or analyzed during the current study are available from the corresponding author upon reasonable request.
