# Supplementary material for: Anti-tumor effects of anti-programmed cell death-1 antibody treatment are attenuated in streptozotocin-induced diabetic mice
Source: Sci Rep. 2023 Apr 12;13:5939. doi: 10.1038/s41598-023-33049-7 (PMC10097709; doi:10.1038/s41598-023-33049-7)
Supplement: Supplementary file 1 — Supplementary Information. [file 41598_2023_33049_MOESM1_ESM.pdf]

Title: Anti-tumor effects of anti-programmed cell death-1 antibody treatment are attenuated in streptozotocin-induced diabetic mice

Masaaki Ito[1], Shintaro Iwama[1], Daisuke Sugiyama[2], Yoshinori Yasuda[1], Takayuki Okuji[1], Tomoko Kobayashi[1], Xin Zhou[1], Ayana Yamagami[1], Takeshi Onoue[1], Takashi Miyata[1], Mariko Sugiyama[1], Daisuke Hagiwara[1], Hidetaka Suga[1], Ryoichi Banno[1,3], Hiroyoshi Nishikawa[2,4], Hiroshi Arima[1]

Affiliation:

[1]Department of Endocrinology and Diabetes, Nagoya University Graduate School of Medicine, Nagoya 466-8550, Japan

[2]Department of Immunology, Nagoya University Graduate School of Medicine, Nagoya 466-8550, Japan.

[3]Research Center of Health, Physical Fitness and Sports, Nagoya University, Nagoya, 464-8601, Japan

[4]Division of Cancer Immunology, Research Institute/Exploratory Oncology Research & Clinical Trial Center (EPOC), National Cancer Center, Tokyo 104-0045, Japan.

Correspondence to:

Shintaro Iwama, M.D., Ph.D.

Department of Endocrinology and Diabetes, Nagoya University Graduate School of Medicine, Nagoya 466-8550, Japan

Phone: (+81) 52-744-2194

Fax. (+81) 52-744-2212

E-mail: siwama@med.nagoya-u.ac.jp

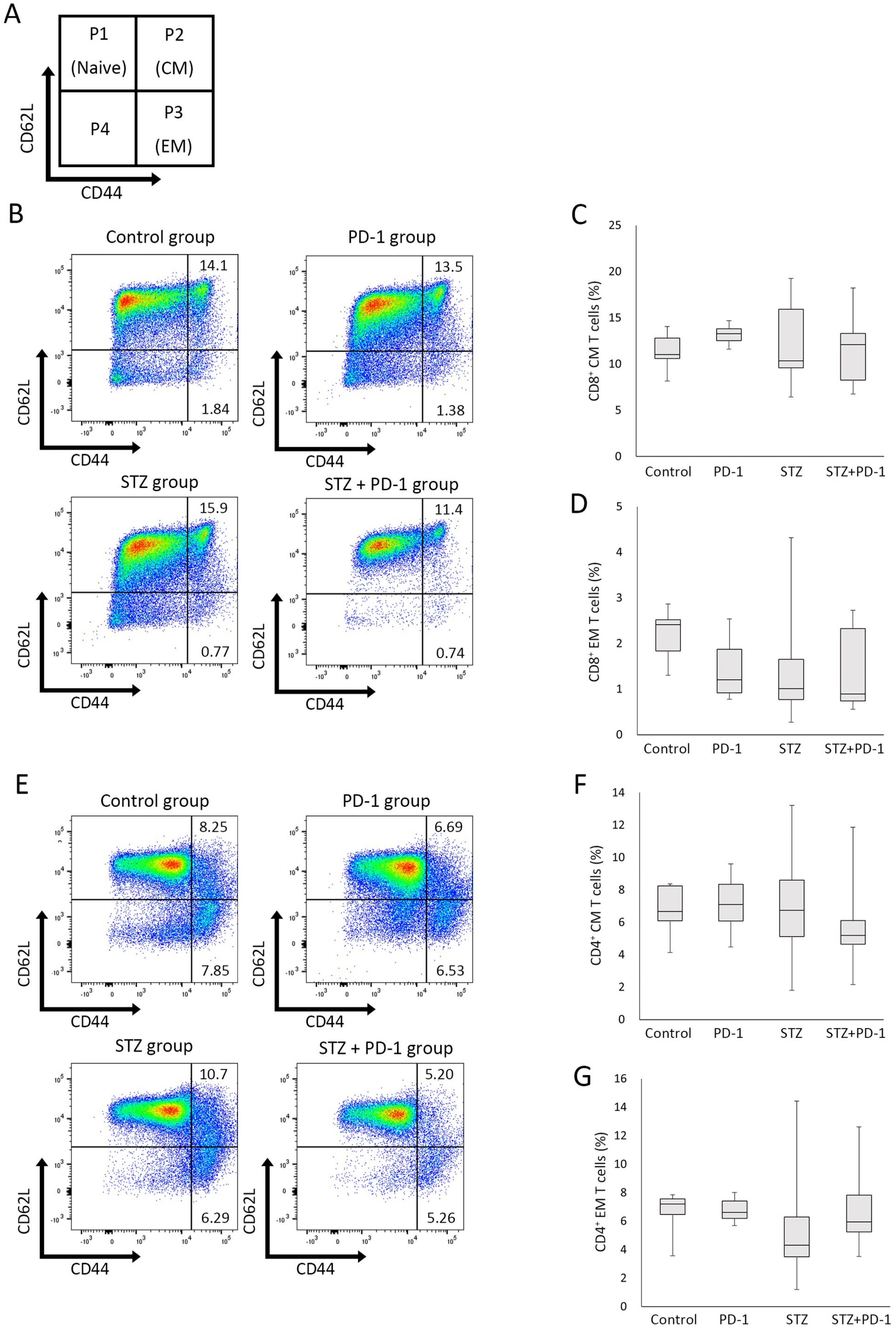

A
P1
(Naive)
P2
(CM)
P4
P3
(EM)
CD62L
CD44
B
Control group
PD-1 group
STZ group
STZ + PD-1 group
14.1
1.84
13.5
1.38
15.9
0.77
11.4
0.74
C
CD8+ CM T cells (%)
D
CD8+ EM T cells (%)
Control
PD-1
STZ
STZ+PD-1
E
8.25
7.85
6.69
6.53
10.7
6.29
5.20
5.26
F
CD4+ CM T cells (%)
G
CD4+ EM T cells (%)

Supplementary Figure S1. STZ-induced diabetes does not change the frequency of memory T cell subsets. (A) Flow cytometry gating for the identification of central memory (CM) T cells and effector memory T cells (EM) in dLNs by differential expression of CD44 and CD62L. P2 (CM T cells); $CD44^{+}$ $CD62L^{+}$, P3 (EM T cells); $CD44^{+}$, $CD62L^{-}$, P1 (Naïve T cells); $CD44^{-}$ $CD62L^{+}$, P4; $CD44^{-}$ $CD62L^{-}$. (B and E) Representative plots of expression of CD44 and CD62L on $CD8^{+}$ T cells (B) or $CD4^{+}$ T cells (E) in dLNs. (C and F) Box and whisker plots of frequencies of CM T cells within the $CD8^{+}$ T cell (C) and $CD4^{+}$ T cell (F) compartments in dLNs. (D and G) Box and whisker plots of frequencies of EM T cells within the $CD8^{+}$ T cell (D) and $CD4^{+}$ T cell compartments (G) in dLNs. On day 14, dLNs were isolated from mice in the control, PD-1, STZ, and STZ+PD-1 groups. Boxes indicate median values with the interquartile range, and whiskers indicate the lowest and highest values. *dLNs: tumor-draining lymph nodes, STZ; streptozotocin, PD-1; programmed cell death-1.*

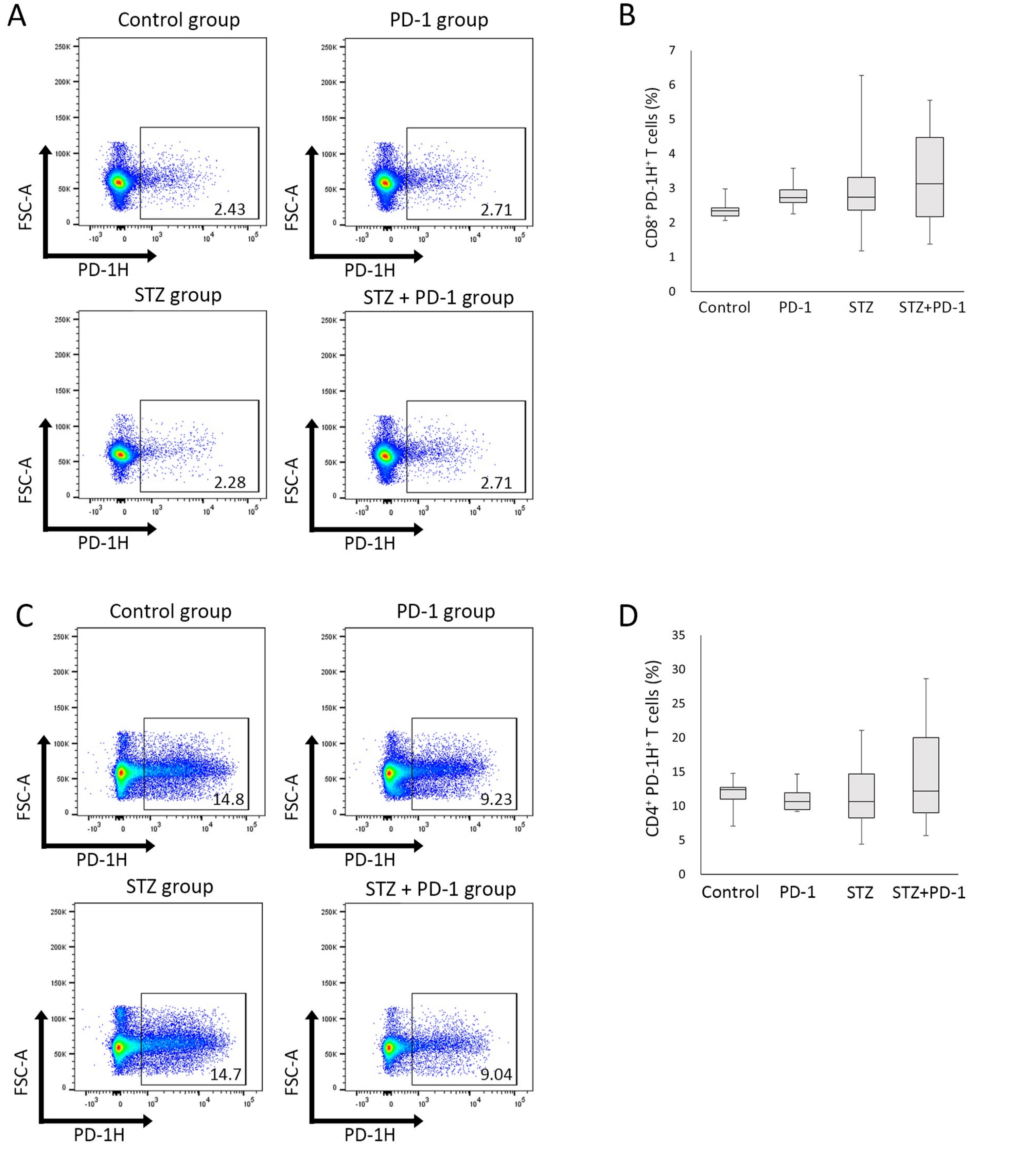

Supplementary Figure S2. STZ-induced diabetes does not change the frequency of PD-1H^{+} cells within the T cell population.

(A and C) Representative plots of the expression of PD-1H on CD8^{+} T cells (A) or CD4^{+} T cells (C) in dLNs.

(B and D) Box and whisker plots of the frequencies of PD-1H^{+} cells within the CD8^{+} T cell (B) and CD4^{+} T cell (D) compartments in dLNs. On day 14, dLNs were isolated from mice in the control, PD-1, STZ, and STZ+PD-1 groups. Boxes indicate median values with the interquartile range, and whiskers indicate the lowest and highest values.

dLNs: tumor-draining lymph nodes, FSC; forward scatter, STZ; streptozotocin, PD-1; programmed cell death-1, PD-1H; programmed cell death-1 homolog.

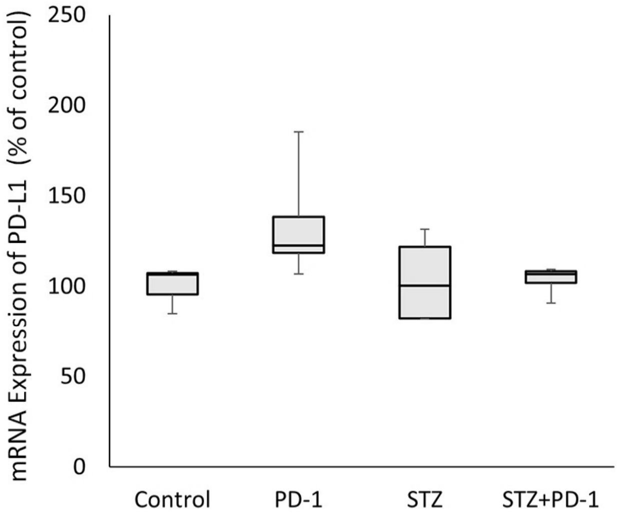

Supplementary Figure S3. STZ-induced diabetes does not change the mRNA expression levels of PD-L1 in tumors.

The mRNA expression of PD-L1 as assessed by qRT-PCR in tumors of mice in the control (n = 3), PD-1 (n = 4), STZ (n = 3), or STZ+PD-1 (n = 4) groups. Boxes indicate median values with the interquartile range, and whiskers indicate the lowest and highest values.

PD-1; programmed cell death-1, PD-L1; programmed cell death ligand 1, qRT-PCR; quantitative real-time reverse transcriptase polymerase chain reaction, STZ; streptozotocin.

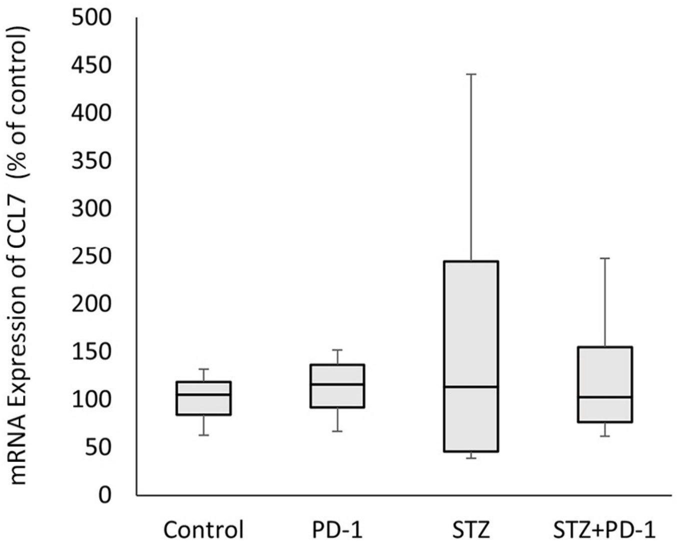

Supplementary Figure S4. The mRNA expression levels of CCL7 in MC38 tumor from STZ-induced diabetic mice.

The mRNA expression levels of CCL7 as assessed by qRT-PCR in MC38 tumor in the control, PD-1, STZ, and STZ+PD-1 groups. Boxes indicate median values with the interquartile range, and whiskers indicate the lowest and highest values.

CCL7; C-C motif chemokine ligand 7, qRT-PCR; quantitative real-time reverse transcriptase polymerase chain reaction, STZ; streptozotocin.

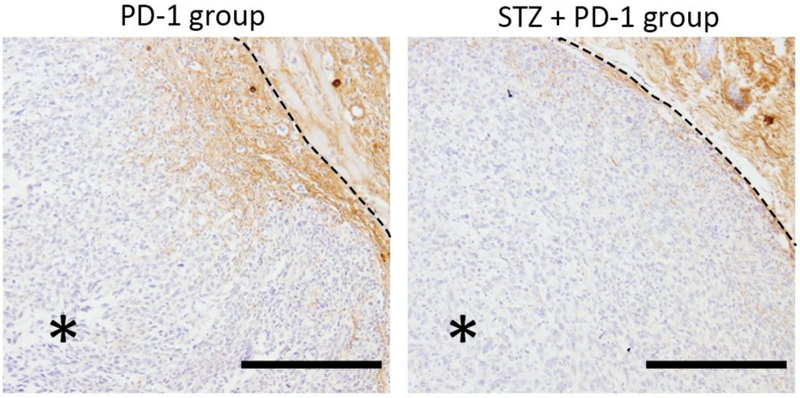

Supplementary Figure S5. Immunohistochemical staining of CD11c$^+$ cells in the MC38 tumors of mice in the PD-1 or STZ+PD-1 groups.

Immunohistochemical staining of CD11c$^+$ cells in the MC38 tumors of mice in the PD-1 or STZ+PD-1 groups. Scale bars, 200 μm. The dashed line indicates the edge of the tumor. *Tumor tissue area.

PD-1; programmed cell death-1, STZ; streptozotocin.

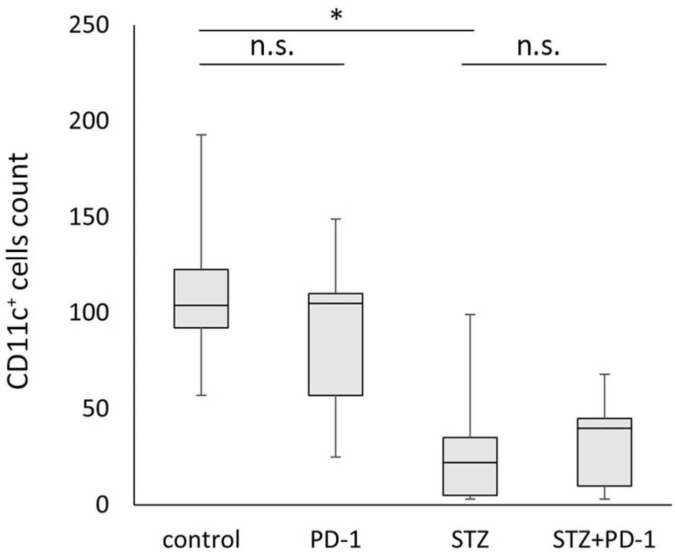

Supplementary Figure S6. The number of CD11c^{+} cells in MC38 tumors were no differences between the control and PD-1 groups and between STZ and STZ-PD-1 groups.

The number of CD11c^{+} cells in MC38 tumors in the control, PD-1, STZ, and STZ+PD-1 groups. Boxes indicate median values with interquartile range, and whiskers indicate the lowest and highest values. One-way ANOVA and Tukey's test are used. *$P < 0.05$.

ANOVA; analysis of variance, n.s.; not significant, PD-1; programmed cell death -1, STZ; streptozotocin.

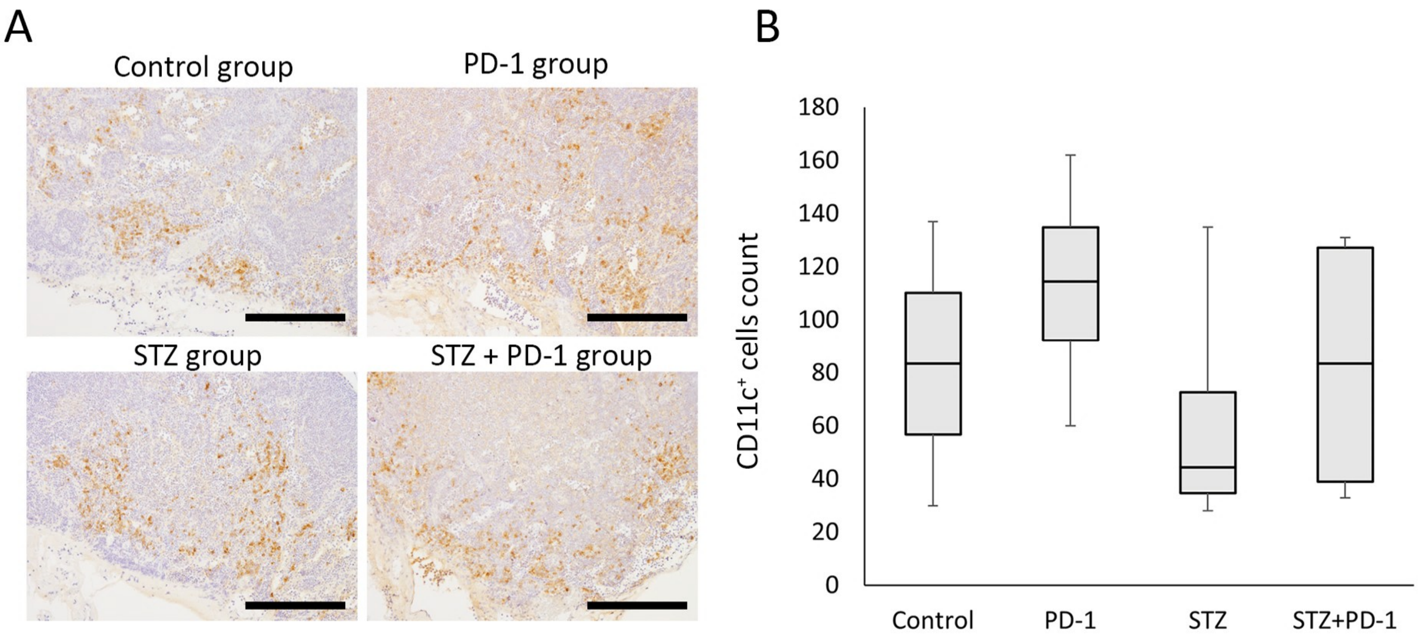

Supplementary Figure S7. The number of CD11c^{+} cells in dLNs were no differences among the control, PD-1, STZ and STZ+PD-1 groups.

(A) Immunohistochemical staining of CD11c^{+} cells in dLNs of mice in the control, PD-1, STZ, and STZ+PD-1 groups. Scale bars, 200 µm. (B) The number of CD11c^{+} cells in dLNs in the control, PD-1, STZ, and STZ+PD-1 groups. Boxes indicate median values with interquartile range, and whiskers indicate the lowest and highest values.

dLNs: tumor-draining lymph nodes, PD-1; programmed cell death -1, STZ; streptozotocin.

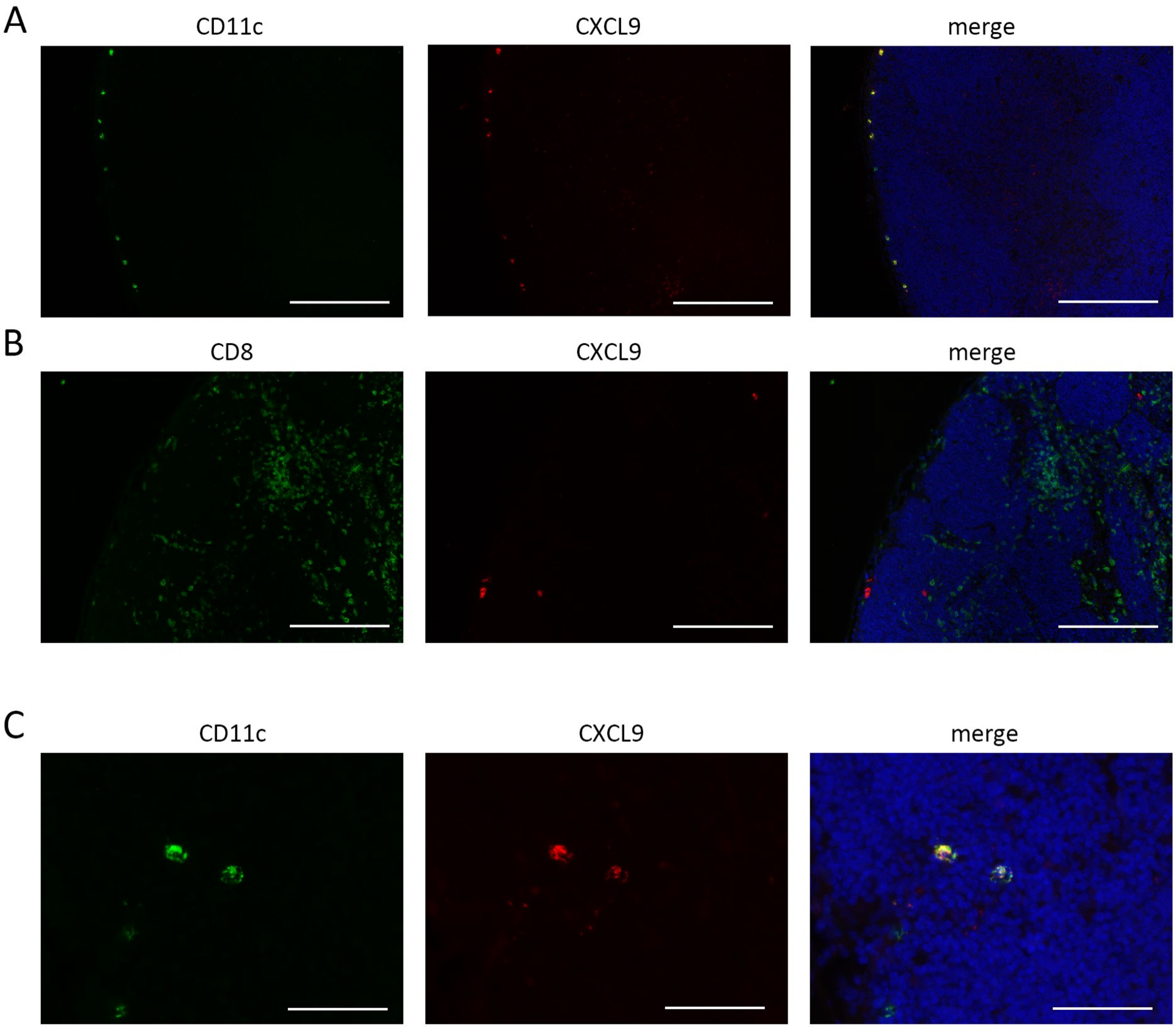

Supplementary Figure S8. Low and high magnification images showing the presence of CXCL9⁺ CD11c⁺ cells and CD8⁺ cells in dLNs.

(A and B) Double immunofluorescence microscopy shows the expression of CXCL9 (red) on CD11c⁺ cells (green) (A) or CD8⁺ cells (green) (B) in dLNs, respectively. Scale bars, 200 μm. (C) Double immunofluorescence microscopy shows the expression of CXCL9 (red) on CD11c⁺ cells (green) in dLNs. Scale bars, 50 μm.

dLNs: tumor-draining lymph nodes, PD-1; programmed cell death -1, STZ; streptozotocin.

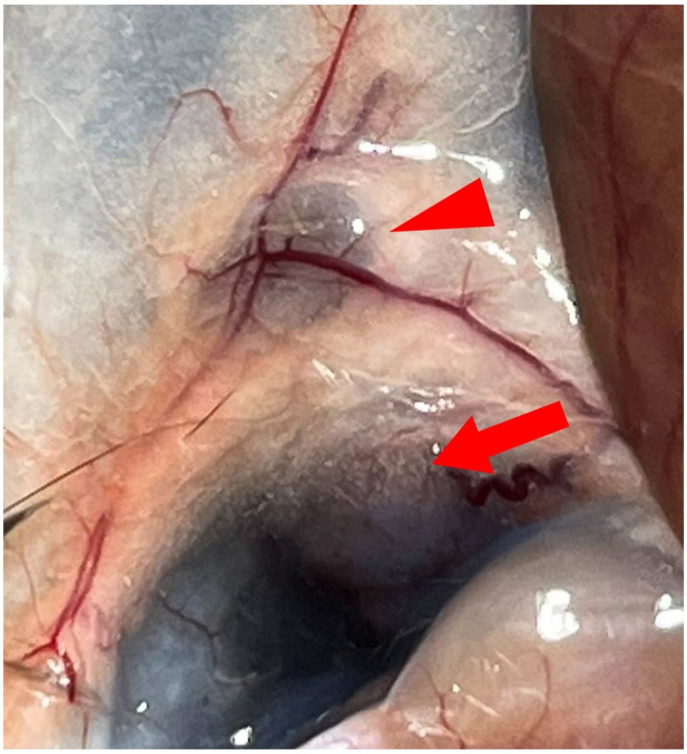

Supplementary Figure S9. Image showing dye flow of trypan blue in a dLN.

Image showing dye flow 24 hours after the injection of 0.4% trypan blue into MC38 tumor. The arrowhead represents a dLN. The arrow represents MC38 tumor.

dLN: tumor-draining lymph node.

gene	Log10 (HG-MC38 $2^{-\Delta Ct}$)	Log10 (LG-MC38 $2^{-\Delta Ct}$)
CCL7	-2.0085	-1.6280
CXCL1	-2.2794	-2.1307
CCL2	-0.5214	-0.3757
CMTM3	-1.3703	-1.2607
CCR10	-2.5774	-2.5160
TLR4	-2.1951	-2.1367
CCL25	-1.9724	-1.9170
CXCL3	-2.6737	-2.6304
CCL8	-2.6347	-2.6123
CX3CL1	-2.3186	-2.3053
MAPK1	-0.8405	-0.8272
HIF-1α	-1.1445	-1.1313
MAPK14	-1.3613	-1.3510
TGF-β1	-1.2619	-1.2697
CMTM4	-1.5750	-1.5888
SLIT2	-1.7526	-1.7996
CXCL10	-0.9398	-1.0620
CMTM6	-1.7707	-1.8989
CCL5	-1.9663	-2.1096

Supplementary Table S1. mRNA expression levels of chemokines and chemokine receptors in MC38 cells cultured in low glucose (LG-MC38) and high glucose (HG-MC38) analyzed by RT2 Profiler PCR Array.

gene	Log10 (Control group $2^{-\Delta Ct}$)	Log10 (PD-1 group $2^{-\Delta Ct}$)
CXCL9	-1.8561	-1.6545
CCR6	-1.3083	-1.1277
CXCL13	-0.0199	0.1306
CXCR4	-0.7243	-0.5858
CXCR5	-1.7538	-1.6183
CCRL2	-2.2927	-2.1782
CXCL10	-1.1788	-1.1006
CCR3	-2.0067	-1.9374
MAPK1	-1.0795	-1.0313
CXCR3	-1.5100	-1.4829
CMTM6	-1.5882	-1.5611
ITGB2	-0.8597	-0.8688
CCR8	-2.3107	-2.3258
HIF-1α	-1.2089	-1.2270
MAPK14	-1.5521	-1.5762
CCR9	-2.4191	-2.4582
TNF	-2.1451	-2.2054
CCR7	-0.6460	-0.7213
CCL4	-2.2927	-2.3679
CCL19	0.0975	0.0163
TGF-β1	-1.0012	-1.0825
CXCL16	-0.8868	-0.9711
CCR5	-1.9465	-2.0338
CCL8	-0.2095	-0.3329
XCL1	-1.6996	-1.8261
CCR2	-1.5521	-1.6906
TLR4	-1.8411	-1.9886
CCL12	-0.9500	-1.1036
CCL22	-1.2059	-1.3896
TLR2	-2.0910	-2.2836
CCL5	-0.0138	-0.2155
CMTM3	-1.2571	-1.4769
ITGAM	-1.9043	-2.1241
CXCL12	-0.8477	-1.0855
CXCR6	-1.6123	-1.8953
IL16	-1.4859	-1.8411
IL1b	-1.9013	-2.2716
CCL17	-1.7689	-2.1421
CCL6	-0.8477	-1.2691
ACKR1	-1.5732	-2.0127
CCL9	-1.8321	-2.2957
PF4	-1.7207	-2.2776
CCL7	-0.8929	-1.6454
CCL11	-1.4498	-2.3198
CCL2	-1.0042	-1.9465

Supplementary Table S2. mRNA expression levels of chemokines and chemokine receptors in tumor-draining lymph nodes of the control group and PD-1 group analyzed by RT2 Profiler PCR Array.

PD-1; programmed cell death-1

gene	Log10 (STZ group $2^{-\Delta Ct}$)	Log10 (STZ+PD-1 group $2^{-\Delta Ct}$)
CCL6	-1.0608	-0.8513
ITGAM	-2.0482	-1.9440
CXCR5	-1.6027	-1.5166
TNF	-2.0542	-1.9832
PF4	-2.2710	-2.2331
CXCR3	-1.4702	-1.4504
CCR8	-2.2439	-2.2240
CCL9	-2.0602	-2.0584
CCL22	-1.1842	-1.1855
CCL2	-1.9428	-1.9440
CXCL12	-0.9133	-0.9175
CMTM6	-1.4281	-1.4323
IL16	-1.2776	-1.2848
MAPK14	-1.5214	-1.5316
TLR4	-1.8525	-1.8658
CCR6	-1.0608	-1.0921
CCL17	-1.9880	-2.0254
CCL19	0.0831	0.0397
CCR5	-2.0151	-2.0675
TGF-β1	-1.0036	-1.0590
CXCR6	-1.7984	-1.8568
CCL11	-1.8044	-1.8808
CCL8	-0.2812	-0.3606
CCR2	-1.6478	-1.7333
CMTM3	-1.4100	-1.4955
CCRL2	-2.0512	-2.1367
CCL7	-1.6207	-1.7093
CCR3	-1.9428	-2.0374
IL1b	-2.3643	-2.4618
MAPK1	-0.9344	-1.0410
ACKR1	-1.5094	-1.6159
TLR2	-2.0422	-2.1578
CXCR4	-0.4919	-0.6075
HIF-1α	-1.0638	-1.1885
CXCL16	-0.8531	-0.9808
XCL1	-1.9037	-2.0584
ITGB2	-0.8501	-1.0048
CCR7	-0.6484	-0.8333
CXCL13	0.1312	-0.0536
CCR9	-2.3311	-2.5160
CCL5	-0.1608	-0.3576
CCL12	-0.9133	-1.1764
CCL4	-2.4486	-2.7238
CXCL9	-1.5756	-1.9170
CXCL10	-0.9525	-1.3330

Supplementary Table S3. mRNA expression levels of chemokines and chemokine receptors in tumor-draining lymph nodes of the STZ group and STZ+PD-1 groups analyzed by RT2 Profiler PCR Array.

PD-1; programmed cell death-1
